# How Personal Values Count in Misleading News Sharing with Moral Content

**DOI:** 10.3390/bs12090302

**Published:** 2022-08-24

**Authors:** Francesca D’Errico, Giuseppe Corbelli, Concetta Papapicco, Marinella Paciello

**Affiliations:** 1 Department of Education, Psychology and Communication, University of Bari ‘Aldo Moro’, 70121 Bari, Italy; 2 Faculty of Psychology, Uninettuno University, 00186 Rome, Italy

**Keywords:** misleading news, moral foundations theory, basic human values, moral emotions, credibility, online sharing

## Abstract

The present study investigates the personal factors underlying online sharing of moral misleading news by observing the interaction between personal values, communication bias, credibility evaluations, and moral emotions. Specifically, we hypothesized that self-transcendence and conservation values may differently influence the sharing of misleading news depending on which moral domain is activated and that these are more likely to be shared when moral emotions and perceived credibility increase. In a sample of 132 participants (65% female), we tested SEMs on misleading news regarding violations in five different moral domains. The results suggest that self-transcendence values hinder online sharing of misleading news, while conservation values promote it; moreover, news written with a less blatantly biased linguistic frame are consistently rated as more credible. Lastly, more credible and emotionally activating news is more likely to be shared online.

## 1. Introduction

**Figure 1:**
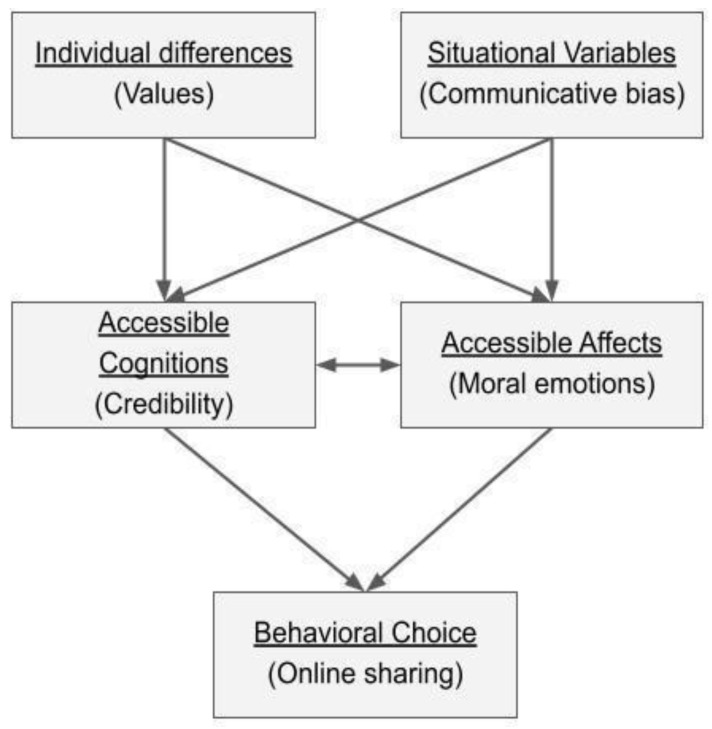
Theoretical framework.

With the advent of social media, misinformation has rapidly spread representing a concrete threat to democratic processes in current digital societies. Indeed, misinformation can “emphasize divisions and erode the principles of shared trust that should unite societies” [1] (p. 81). Misinformation can be defined as false, misleading, or inaccurate content shared to produce a particular judgment in message recipients, irrespective of the veracity (or bias) of what is shared [2]. It is differentiated from the term “disinformation”, which is deliberately deceptive [3]. Despite the fact that false, misleading, and distorted news is well recognized as one of the major concerns in contemporary societies [4], it remains unclear how different personal factors can co-occur and interact to foster this deleterious phenomenon. In the present study, we focus on misinformation concerning some particular topics, such as moral violations in different domains [5,6]. Specifically, we adopt an interactionist approach to investigate the interplay among individual psychological dimensions (i.e., personal values, affective and cognitive processes) and online communicative factors (i.e., moral contents and source biases), leading to the online sharing of what we have appointed “moral misleading news” (Figure 1). We believe that the integration of the communicative and individual perspectives could be useful to better understand why, how, and by whom different kinds of “moral misleading news” could be shared.

Specifically, with regard to individual dimensions, we focus on “distal” pre-existing values [7] and “proximal” cognitive and affective processes [8,9] activated by moral misleading news. The literature has already suggested that the spread of false news could be due to goal-oriented motivations, such as partisan orientation [10]. However, the majority of studies have mainly focused on political orientations as possible motivational determinants of: (a) distortion [11], (b) spreading of disinformation [12], and (c) increasing online polarized and uncivil discussions [13]. In line with this kind of literature, we aim to understand the role played by those specific goals that orient individual cognitive processing over and above political orientations, namely personal values [14]. Personal values are strongly linked to cognitive and emotional processes activated in a specific situation. Thus, distal values can consistently affect the proximal individual responses that, in turn, influence misleading news sharing. In particular, following the theoretical considerations of Jost and colleagues [15] regarding shared reality and conformity, moral values can be considered good predictors of misleading news sharing. As suggested by the literature on misinformation [16], we consider particularly relevant as possible mediators between moral values and online sharing behavior those cognitive and affective processes related to credibility and moral emotions.

With regard to online communicative factors, in the present study, we consider separately the formal aspect of misleading news and its content. To this end, in order to consider the effects of formal information manipulation, we adopt a distinction between the way in which specific content is framed through a high or low level of bias generally found in misleading news [17,18]. Conversely, to evaluate the effects of content on online sharing, we examine moral communications by relying on moral foundation theory [5,6] for two main reasons. First, in line with the literature, we observe that personal values and moral domains are strongly correlated [19]. Thus, it is plausible that some content is more activating for some individuals than it is for others. Secondly, we test with a more comprehensive theoretical framework the activating function of some sensitive news concerning moral and ethical issues. Moral topics might then trigger emotional responses and confirmatory reasoning in a consistent manner; again, we hypothesized that affective and cognitive processes might act as mediators in the relationship between content and online sharing of moral misleading news.

To verify this, based on this interactionist approach, we carried out a quasi-experimental study. The aim is to understand the effect that two different communicative frames (low vs. high level of bias) jointly with personal values for each of the five moral domains (Care, Fairness, Loyalty, Authority, and Purity) have. The effect should be on the activation of moral beliefs and emotions, which, in turn, mediate the influence of both individual and communicative factors on the sharing of online misinformation. Hence, the research questions from which the research proceeded are:

- RQ1: What is the effect of news framing on sharing behavior?
- RQ2: What is the role of credibility and moral emotions in sharing moral news?
- RQ3: How do personal values affect news sharing across the moral domains?

### 1.1. The Individual Dimensions of Misinformation

The psychological approach for the misinformation processes’ understanding has mainly indicated how people fall victim to fallacious and misleading news when they rely on their “lazy” cognitive processes [20] or also their motivational factors, such as the desire for entertainment or fear of missing out [21,22]. In particular, according to the motivated reasoning account [23], people use their reasoning skills to protect and support their identity, their ideologies, and beliefs rather than to obtain authentic information [24]. Therefore, intentions and behaviors are strongly favored by their attitudes toward certain ideas or ideologies, prompting people to over-believe content consistent with their position. In contrast, they become skeptical when they process content that is inconsistent with them as a kind of “identity-protective cognition” [24]. In this light, to our knowledge, even if largely theorized [15], few works have experimentally tested the role played by the motivational factor exerted by personal values.

A comprehensive definition of values is “trans-situational goals that vary in importance and serve as guiding principles in the life of a person or a group” [25]. As motivational determinants, they can be useful to understand how individuals differ in their perception, interpretation, and behavioral decision in the face of the same situation/stimulus. Consequently, values can also be individual dimensions that can contribute to the credibility and sharing of fake news, following the “motivated reasoning theory” [26] that indicates how we can give more credibility to something that we feel is close to our principles. Values can be the basis for individual evaluations. Objects are positively evaluated if they are in line with personal values; in contrast, they are negatively evaluated if they hinder or threaten their attainment [7]. Moreover, since values can affect people’s beliefs that people hold about how the world functions [7,27], they can affect the grade of credibility of some events and objects that are differently consistent with one’s world view.

Among the theorists that have studied personal values, one of the most prominent values researchers is Shalom Schwartz [25], who has proposed a taxonomy based on the fundamental social and biological needs of human beings. Personal values can be mapped into a circumplex model defined by two dimensions: one dimension captures the conflict between values that emphasize the independence of thought and action, and proneness for change (i.e., openness to change); and values that emphasize preservation of group and social order, and resistance to change (i.e., conservation values). The other dimension captures the conflict between values that emphasize concern for the welfare and interests of others (i.e., self-transcendence) and values that emphasize the pursuit of one’s interests and relative personal success and dominance over others (i.e., self-enhancement). Clearly, many values are moral values but not all of them have an intrinsic moral nature. Moral values are related to welfare and fairness concerns (e.g., benevolence, patriotism, and traditions), and are related to two basic universal human needs: (1) the need of protecting one’s own group from internal and external threats and (2) the need of fostering reciprocal exchange [28]. More in-depth, according to Schwartz’s theory [25], the “Conservation” values (i.e., conformity, tradition, and security) have the function of creating the internal cohesion necessary to face possible threats by “binding” the group together; whereas “Self-transcendence” values (i.e., benevolence and universalism) have the function of promoting prosocial bonds between individuals—even with others outside the group. Framing in Haidt domains [29,30], the “Conservation” values concern Ingroup, Authority, and Purity (e.g., national security, obedience, conformism), the “Self-transcendence” includes the main values related to the Harm and Fairness foundations (e.g., social justice and right to protection). Concerning public opinion, several studies have attested that values orient attitudes toward policy views and electoral choices [31,32,33]. For example, conservation values predict support for the right political orientation, and self-transcendence for the left political orientation [28].

Motivational factors can be considered as strictly associated with emotional and cognitive factors in fake and misleading news credulity and sharing [34]. In particular, the most influential position is that emotions (regardless of the specific type) play a predictive role of increased confidence in misleading news, lowering the ability to discern between true and false news [35]. Therefore, the use of emotions increases trust in hoaxes; the more individuals rely on emotional activation, the more they perceive false or misleading information as accurate. This can explain why false news is definitely conceived to activate individuals’ emotional responses by leading them to be more credible [35]. In this perspective, the failures in the identification processes of credible news would derive from a combination between the lack of analytical thinking and strong emotionality.

In addition, Pennycook and colleagues [36] highlighted how affective processes can play a pejorative role in credibility, whether heightened emotionality is associated with increased belief in fake news. Moreover, Pennycook and colleagues [36] state that emotion, both positive and negative, is predictive of fake news credulity and that it increases the possibility of discerning real from fake news. Specifically, the authors study the role of “momentary” [36] (p. 4) emotions, those emotions that are activated during the reading of news, and credibility. The results suggest that momentary negative emotions diminish people’s ability to discern between real and fake news.

The results relating to how the emotional aspects lead to greater credulity on the part of social media users, however, are based on news mainly politically connoted, since the assumption that moves them is precisely that, referring to an activation close to in-group favoritism [37] or the desire for conformity [15]. However, to fully understand the contribution of personal values, it can be useful to deepen the role played by two crucial factors: the individuals’ value orientation and the moral domain of the news. Concerning personal values, among the moral ones postulated in Schwartz’s model [25], that are self-transcendence and conservation, conformity seems to be more crucial for the credibility processing of information [15].

Moreover, since the well-known literature on misinformation [17,38] focused mostly on misleading news content framed with polarized political belonging (e.g., conservative vs. liberal frame), it seems necessary to them to broaden the focus by concentrating the attention on more inclusive moral domains, which, according to Feldman’s and colleagues [19] studies, are closely related. Their findings suggested, in fact, that self-enhancement values would be associated with generally lower importance for all moral foundations, self-transcendence would mainly be associated with harm–care and fairness–reciprocity moral concerns, conservation would be associated with authority–respect, while openness to change would be associated with high moral relativism, seeing morality as more flexible based on the context.

### 1.2. Communicative Dimensions of Misinformation

When we consider the communicative side of the literature on misinformation, we mainly encounter two aspects of the issue: the content and linguistic form. As to the first, it is well known that content related to the political field typically induces polarization and, in terms of credibility, a certain type of congruence to the readers’ political orientation [38]. Nevertheless, in the field of moral psychology and moral communication, five moral domains have been defined [6,29] that can settle issues concerning moral concerns, and that can activate automatic, relatively effortless, and rapid moral judgments on questions concerning people’s evaluation of well-being, justice, group, religious, or institutional belonging. Specifically, Haidt and his collaborators [5,29,39] identified five moral domains, which are: harm/care, related to human sensitivity to others’ needs and distress, with the function of activating human urges to care for and protect vulnerable individuals; fairness/reciprocity, related to human attentiveness toward equity and reciprocity behaviors that promote collaborative and altruistic social interactions; ingroup/loyalty, related to the human tendency to build cohesive coalitions that compete to obtain restricted resources in order to obtain collective benefits; authority/respect, related to the tendency to generate social hierarchies within which authority is respected and seen as legitimate for maintaining social order; and, lastly, purity/sanctity, related to social practices preventing those contaminants that threaten the survival of the group. The five domains cluster under two higher-order groupings: (1) the “individualizing” foundations (care and fairness) focus on the rights and welfare of the individual; and the “binding” domains (loyalty, authority, and sanctity) focus on the cohesion of the group [29,40].

More recently, social media studies have highlighted that moral contents associated with emotions can become viral. In particular, Brady and colleagues [9] proposed the MAD model (motivation, attention, and design) that indicated how: (i) people are motivated to share moral contents on the basis of their social identity (motivation); (ii) moral contents are better able to capture people’s attention and to involve them than other types of content (attention); and (iii) social media design and affordances (anonymity or social feedback) facilitate and amplify moral content (design). These three factors led to a greater probability to share online moral content, allowing the so-called “moral contagion” [41].

Specifically, the MAD model pointed out that the final decision to share news with moral content would depend on the interaction between psychological factors and environmental factors in social networks. Specifically, the factors considered concern group-based motivations, how moralized stimuli engage our attention, and how the design of social media amplifies these elements.

In this vein, according to the MAD model, it is possible to assume that moral contents activate moral emotions because they are associated with group identity. In other words, in the presence of moral content, emotions, defined as moral (e.g., outrage), tend to strengthen the membership of their group. The structural basis of this social identity [37], which organizes membership in the group, is represented by personal values. Thus, the motivation that led people to process and share online content can be strictly linked to values, especially when misleading news describes moral violations.

As regards the role played by the formal and stylistic aspect of misleading news, Rashkin, Choi, Jang, Volkova, and Choi [42] found that misleading news more often includes exaggerations (e.g., superlatives), biases, subjectivity, and hedged language than verified news [42]. Frequent biases in misleading news are sensationalism, the presence of high emotionality [17], the discredit of the responsible [18], or also the so-called “factual bias” [43], which consists of the description of one side instead of giving a complex and articulated framework of the news [44].

Recent studies highlighted that news sensationalism led people to trust and fall into misinformation compared to neutral style, especially when they feel a state of uncertainty, and also when they used alternative media to inform [45]; others instead highlighted how the effect of biased news can decrease when people are induced to evaluate news critically [46].

Hence, a suspect source might decrease the perceived credibility of misleading information and this is also confirmed by the research on the “nudge” effect [47] that indicated that, when social media users were asked to assess “the accuracy of headlines”, this activates a kind of “accuracy mind set” [44] that makes people less credulous and more critical toward the biased headlines. Hence, we focused on subjectivity and sensationalism and investigated whether these patterns affect individuals’ credibility evaluations of online misinformation articles.

Sensationalism and the perception of credibility, however, do not fully explain the users’ sharing behavior of misleading news. More recent studies [20] on sharing behavior, in fact, show that people were apparently willing to share content that they could have identified as being inaccurate. In this study, in fact, participants who were asked about the accuracy of a series of titles rated the real titles as much more accurate than the fake titles but, when asked if they would share the titles, veracity had little impact on shared intentions in the context of policy titles [20]. As a result, sharing intentions for fake titles were much higher than evaluations of their truth (for example, more than 91%). As a result, the sharing of low-quality news content on Facebook is associated with the ideological extreme; in fact, in these cases, political motivation prevails [48] and ideological concordance is a much stronger predictor of sharing than it is of faith [20]. In this regard, research has shown that people who share fake news on social networks are, for example, those with a populist ideological orientation, with a strongly conservative ideological orientation, and with an age of over 65 [49].

## 2. Method

### 2.1. Participants

Volunteers who agreed to take part in the quasi-experiment were recruited through the researchers’ two referring faculties (Psychology and Communication Studies), and received and completed one of the two questionnaires required by the procedure for a total of 132 participants; of these, two responses were excluded from the analysis due to being incomplete (98.5% completion rate).

The final sample consisted of 45 males, 83 females, and 2 non-binary/other (younger people, 17–30: 47.7%, M = 21.5, SD = 3.1; young adults, 31–50: 23.1%, M = 32.0, SD = 4.1; adults, 50+: 29.2%, M = 53.3, SD = 6.7). After an appropriate explanation of the methods, timing, and procedures of the study, all students personally provided consent if they were of age, whereas consent was given by parents if the participant was underage at the time of the observation.

### 2.2. Procedure

Participants were asked to accurately read the proposed stimulus, i.e., a screenshot of a news item found online, and immediately afterward to answer self-report questions regarding their cognitive assessments of the veracity and their emotional state in relation to the news item they had just read, as well as regarding their intention to share it on social media. This procedure was repeated five times (one for each news story based on the five different contents), while each subject was randomly assigned to one of two groups associated with a different questionnaire. The first group (*N* = 57) encountered five news screenshots written in a dry style and with a low bias, while the second group (*N* = 73) was engaged with the same news story contents, but presented following an emotionally activating communicative framing (i.e., a high linguistic bias). In each of the two cases, completion of the full battery took 30 to 50 min. The experimental design was mixed, with a within-subjects independent variable (i.e., the five moral contents) and the type of bias (subtle or blatant) between subjects. The presentation of the specific stimuli, items, and data collection took place through two different questionnaires, the first containing the five news screenshots written following an emotionally activating communicative framing, while the second one had the same pieces of news presented with a dry and impartial style.

Before administering the measures and during the data collection, the final purpose of the study was outlined and participants were informed that their participation in the study was voluntary, reminding them that they were completely free to refuse to participate or withdraw from the study at any time without consequence. None of the participants refused to participate or withdrew from the study, which was conducted between February and June 2021. All the procedures followed the Helsinki ethical principles and ethical codes of AIP (Italian Psychology Association) and the study was approved by the ethical committee of the university of one of the co-authors. Informed consent was obtained for all participants.

### 2.3. Measures

#### 2.3.1. News Stimuli

For this study, 10 artificial screenshots of posts on Instagram from a hypothetical online news page were created ad hoc on the basis of moral violation scenarios validated by Clifford et al. [50]. These screenshots were reproduced as faithfully as possible in order to replicate the way of information exchange through screenshots, which is widely used among young people [20,51,52]. The news items have been artfully designed to activate the content domain for each of the five moral foundations outlined by Haidt et al. [29], namely: Care, Fairness, Loyalty, Authority, and Purity. It is because of the large individual differences in the salience of these basic human motivational modules that we decided to include in the quasi-experiment all five misleading news stories. Each of the five moral domains has been declined through two different screenshots, changing in pairs the linguistic modality of presentation of the news. In the second condition, the communicative framing adopted is highly biased, emotionally activating, and judgmental, similar to what happens in the widespread online misinformation outlets. By contrast, in the first condition, the communicative framing style is less biased and judgmental. Moral misleading news biases were coded 1 (subtle bias) or 2 (blatant bias). The image presented in the Instagram post is the same in the two alternatives, differing according to the five moral domains in such a way as to simulate a real screenshot obtained from a comparable news item found online.

**Figure A1:**
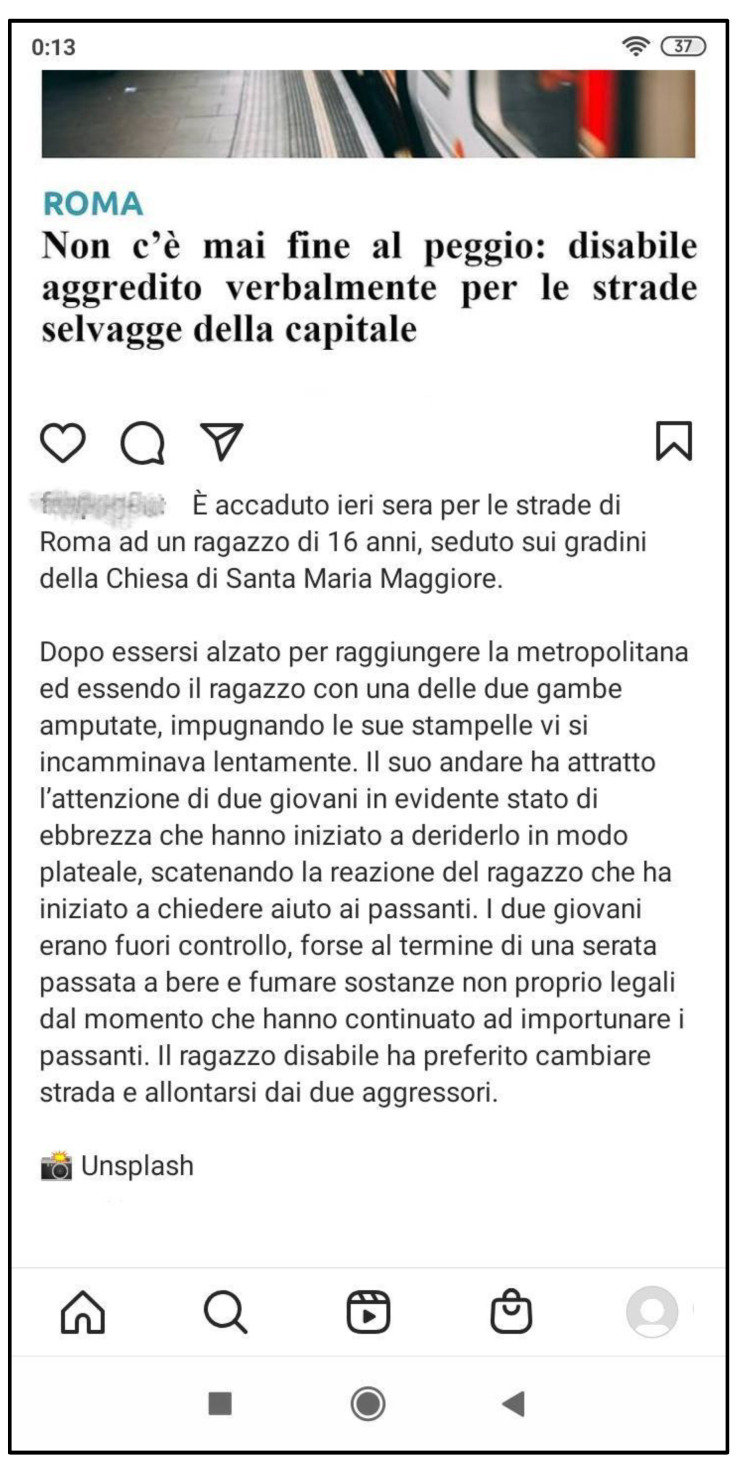
Example of stimulus for the domain of Care: blatantly biased condition.

**Figure A2:**
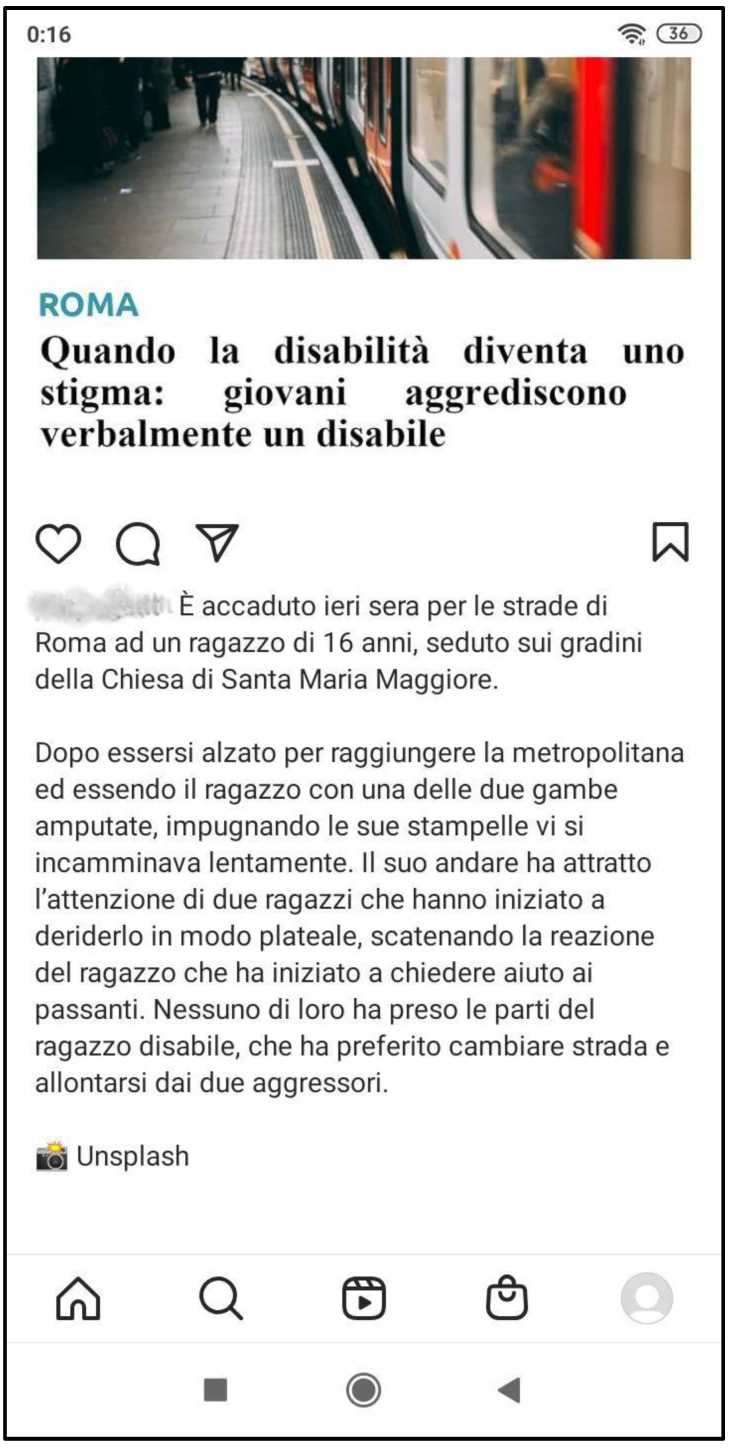
Example of stimulus for the domain of Care: subtly biased condition.

As an example, the screenshot related to the moral domain of Care for the first linguistic modality and the second modality are presented in Appendix A (Figure A1 and Figure A2).

#### 2.3.2. Intention to Share

The subject’s intention to share the news item presented with the screenshot was investigated through a direct single-item, closed-ended question, “Would you share this news item on social media?” The five response options from which the respondent could choose ranged from “Absolutely not” to “Absolutely yes”. This question was prompted immediately after the presentation of each of the five screenshots related to the five moral scenarios.

#### 2.3.3. Cognitive Evaluation of the Veracity of the News Item

This ad hoc scale included 12 items aimed at assessing the cognitive evaluation concerning the piece of news contained in the screenshot. For each item, participants rated their agreement or disagreement with the presented evaluation on a 5-point scale (“very little” = 1; “very much” = 5). For instance, two sample items of the scale are: “In your opinion, how credible is the news you read?” and “In your opinion, how truthful is the news you read?” Among the items to which the subjects responded, those referring solely to the subscale of credibility of the news were selected for the present study, i.e., the perceived credibility, truthfulness, and reliability ratings of the news. Cronbach’s alphas for the credibility-related items were 0.86, 0.93, 0.93, 0.94, and 0.95 for the five moral domains of Care, Fairness, Loyalty, Authority, and Purity, respectively, showing an average excellent degree of reliability.

#### 2.3.4. Evaluation of Moral Emotions

This scale measured the emotional response (“What emotions did you feel while reading the news?”) following the presentation of the news screenshot stimulus, investigating a wide range of both positive and negative, activating and deactivating emotions. “Anger” and “enthusiasm” are sample items, to which participants responded via a 5-point Likert scale (“very little” = 1, “very much” = 5).

Many of these items had zero variability, receiving a score of 1 from all respondents—thus indicating the complete absence of the corresponding emotional reaction elicited by the scenario presented. Almost all of the emotions most frequently reported by the subjects were indeed moral emotions, defined by Haidt et al. [29] (p. 110) as those “linked to the interests or welfare either of society as a whole or at least of persons other than the judge or agent”. Consistent with the moral nature of the scenarios proposed, the emotions selected for the present study were, therefore, those that have been shown to provide the motivational drive to do what is considered right and avoid doing what is considered wrong [53]: disappointment, disgust, pity, anger, shame, fear, compassion, anger, discouragement, concern, anxiety, embarrassment, sadness, and contempt. Cronbach’s reliability coefficients for the moral emotion scale for the five moral domains were 0.91 (Care), 0.91 (Fairness), 0.92 (Loyalty), 0.95 (Authority), and 0.93 (Purity), indicating for each case an excellent degree of reliability.

#### 2.3.5. Value Dimensions of Self-Transcendence and Conservation

To assess the value salience of the respondents, the Italian translation of the original 40-item Portrait Values Questionnaire was used [54]. Being highly relevant to the scope of this research, we decided to take into account the two higher-order dimensions of Conservation (incorporating Tradition, Security, and Conformity) and Self-Transcendence (which includes Benevolence and Universalism) from the original 10 empirically derived values along the two orthogonal axes of the circumplex model [55]. Cronbach’s alphas for the two subsets of values pertaining to Conservation and Self-Transcendence were 0.78 and 0.86, providing, respectively, an acceptable and a good degree of reliability.

## 3. Results

Table 1 summarizes the descriptive statistics for the relevant variables, while Table 2 shows their correlation matrix. Regardless of the moral domain considered, the credibility of a news item was positively correlated with its sharing intention (Care: r = 0.467, *p* < 0.01; Fairness: r = 0.330, *p* < 0.01; Loyalty: r = 0.200, *p* < 0.05; Authority: r = 0.354, *p* < 0.01; Purity: r = 0.490, *p* < 0.01), and also emotional activation showed a significant positive correlation with sharing intention across all domains (Care: r = 0.461, *p* < 0.01; Fairness: r = 0.428, *p* < 0.01; Loyalty: r = 0.562, *p* < 0.01; Authority: r = 0.363, *p* < 0.01; Purity: r = 0.471, *p* < 0.01). Regarding the impact of individual distal dimensions, the interactions turned out to be more complex according to the domains considered, and the increase in news bias was correlated with the decrease in credibility and emotional activation significantly in all domains except for the moral foundation of Loyalty, where the correlation with moral emotions was in the opposite direction (r = −0.175, *p* < 0.05), and in the case of Authority, where no significance was found in the relationship between moral emotional activation and news bias. For each of the five moral domains, path analyses were performed to examine the relationships among cognitive-affective processes triggered by fake news leading to sharing behaviors. Furthermore, in each model, conservation and self-transcendence were defined as latent dimensions using their reference values, which are tradition, security, and conformity in the case of conservation, and benevolence and universalism in the case of self-transcendence. In addition, due to heterogeneous distribution of the sample, gender and generations were included in the model as control variables.

All five models showed an adequate fit to the data and different relationships among the variables under study.

**Figure 2:**
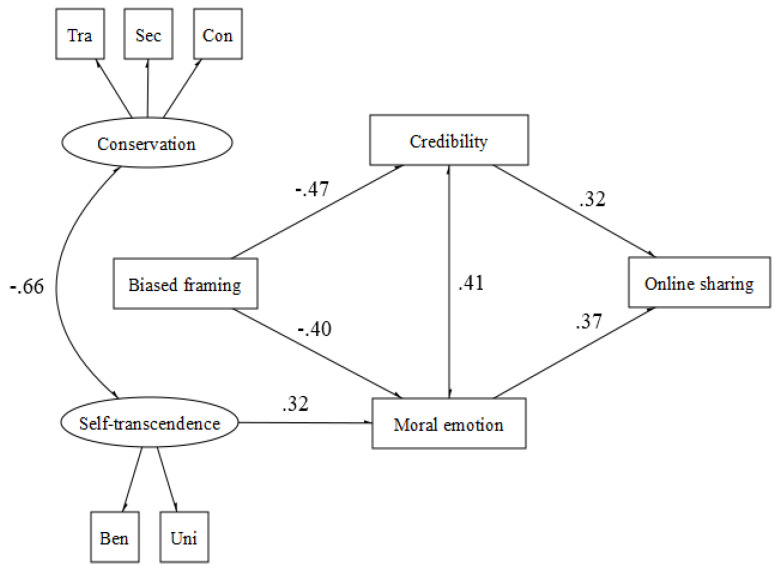
Care.

In depth, with regard to the moral domain of care (χ^2^ (29, N = 130) = 35.504, *p* < 0.001, CFI = 0.94, TLI = 0.93, RMSEA = 0.042 (90% CI = 0.039−0.045) *p* = 1.00, SRMR = 0.048 ≥ 0.18; CFI = 0.98, RMSEA = 0.042 (90% CI = 0.000−0.083) *p* = 0.58, SRMR = 0.046), the results suggest that misleading news presented in a drier communicative style promotes both credulity and moral emotional reactions, which, in turn, influence the likelihood of sharing it. In addition, self-transcendence values promote moral emotional reactions (Figure 2).

**Figure 3:**
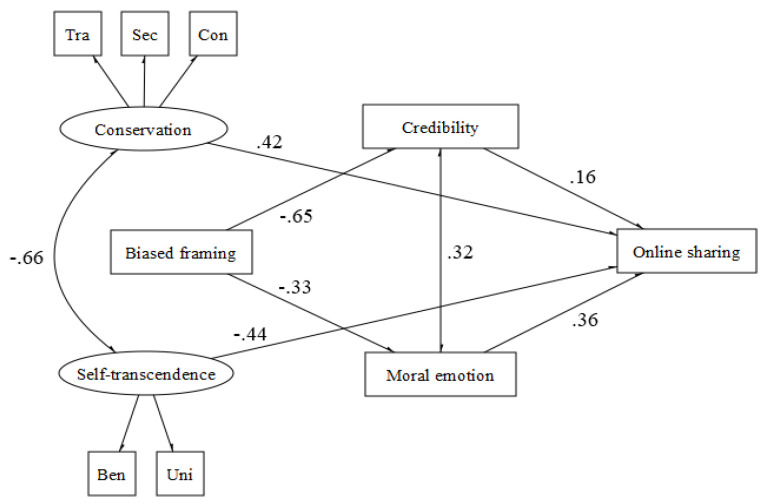
Fairness.

Concerning the fairness domain (χ2 (29, N = 130) = 53.50, *p* < 0.001, CFI = 0.94, TLI = 0.93, RMSEA = 0.042 (90% CI = 0.039−0.045) *p* = 1.00, SRMR = 0.048> 0.003; CFI = 0.93, RMSEA = 0.08 (90% CI = 0.045−0.114) *p* = 0.07, SRMR = 0.052), it is possible to appreciate the same cognitive-affective paths, but values directly influence the sharing behavior. Indeed, conservation values promote sharing behavior, whereas self-transcendence values hinder it (Figure 3).

**Figure 4:**
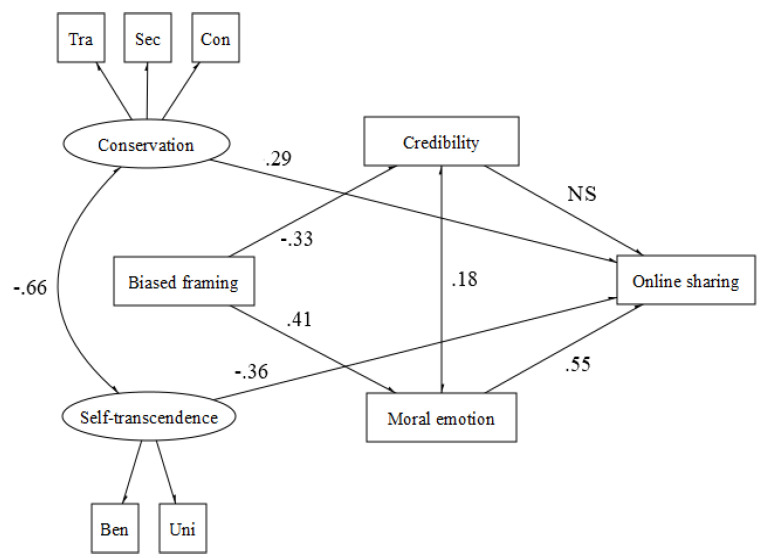
Loyalty.

In the case of loyalty (χ^2^ (29, N = 130) = 33.034, *p* < 0.001, CFI = 0.94, TLI = 0.93, RMSEA = 0.042 (90% CI = 0.039−0.045) *p* = 1.00, SRMR = 0.048 > 0.276; CFI = 0.99, RMSEA = 0.033 (90% CI = 0.000−0.077) *p* = 0.69, SRMR = 0.04), it is interesting to note that news pieces written following an emotionally activating communicative framing foster only affective processes (i.e., moral emotions) that, in turn, positively affect sharing behavior. Moreover, as in the fairness domain, conservation values promote sharing behavior, whereas self-transcendence values prevent it (Figure 4).

**Figure 5:**
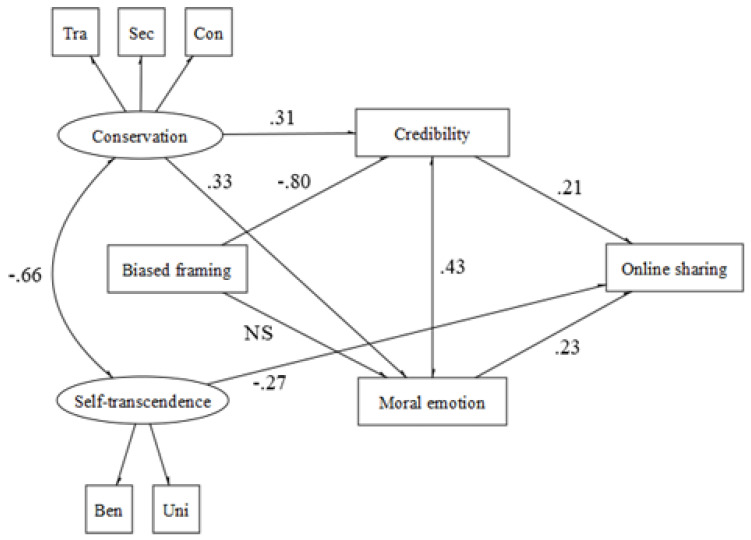
Authority.

With regard to the authority domain (χ2 (29, N = 130) = 31.07, *p* < 0.001, CFI = 0.94, TLI = 0.93, RMSEA = 0.042 (90% CI = 0.039−0.045) *p* = 1.00, SRMR = 0.048 > 0.314; CFI = 0.99, RMSEA = 0.029 (90% CI = 0.000−0.075) *p* = 0.72, SRMR = 0.04), misleading news presented with a dry style foster only credulity that, in turn, affects sharing behavior. Sharing behavior is also positively related to moral emotions and negatively related to self-transcendence values. In addition, in this case, conservation is positively related to both affective and cognitive processes (Figure 5).

**Figure 6:**
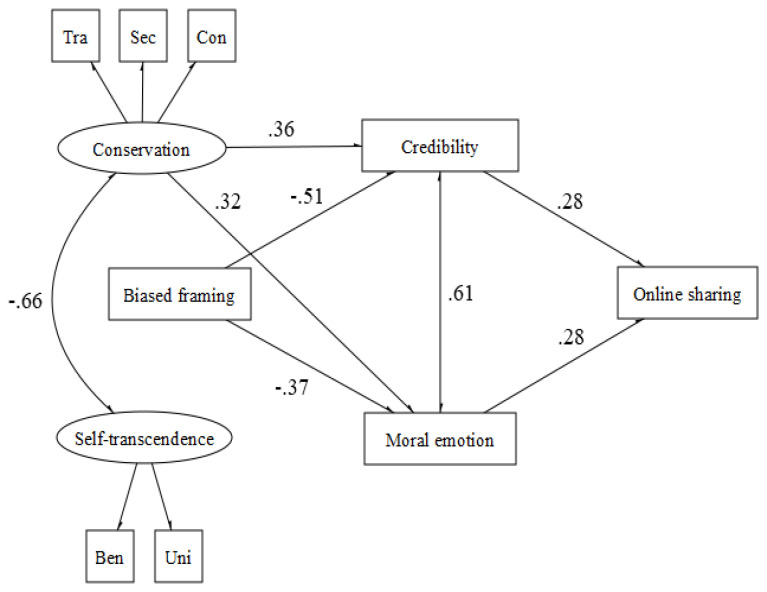
Purity.

With regard to the purity domain (χ2 (29, N = 130) = 31.239, *p* < 0.001, CFI = 0.94, TLI = 0.93, RMSEA = 0.042 (90% CI = 0.039−0.045) *p* = 1.00, SRMR = 0.048> 0.354; CFI = 0.99, RMSEA = 0.024 (90% CI = 0.000−0.073) *p* = 0.76, SRMR = 0.04), news screenshots presented with a subtly biased style foster both credulity and moral emotional reactions that, in turn, affect the probability to share them. As in the moral domain of authority, conservation is positively related to both affective and cognitive processes (Figure 6).

With regard to covariates, in the case of care and loyalty domains, credulity is significantly and negatively associated with generation (the younger are more prone to believe fake news than the older). Moreover, for the care domain, credulity is also associated positively with gender (females are more prone to believe), whereas, for the purity domain, gender is negatively associated with moral emotions (the males emotionally react more than the females).

## 4. Discussion

The results of the present study on the intention of sharing moral misleading news show that specific moral communications activate differentiated cognitive and emotional processes. In general, the results show that the more people believe in the news veracity, the more they get emotionally activated and, consequently, share the news. This is especially true in the case of less biased stimuli. In this case, a low biased stimulus elicits a positive cognitive assessment of truthfulness and subsequent activation of moral emotions; this shows how emotions and beliefs are strictly interconnected.

In other words, differently from some previous works [20] that observed how emotions can be activated mainly in relation to the news falseness, in our case, the emotional activation is parallel to the cognitive evaluation of news veracity across almost all of the five moral domains. As a result, news sharing is influenced by this cognitive-emotional process that intervenes between the presentation of moral misleading news and the subsequent sharing behavior. Therefore, from our results, it emerges that the news is not shared passively, but it is shared only if it is considered credible and emotionally engaging. This can be due also to the fact that, after reading the news, participants were induced to evaluate its veracity; this procedural request can activate a kind of “accuracy mindset” [44] that generally makes people less credulous and more critical toward the biased news, as also demonstrated by the so-called “nudge research” [20]. In addition, our participants were selected on the basis of their linguistic and communicative background, being university students of psychology and communication science.

Considering four domains, i.e., care, fairness, authority, and purity, misleading news presented with a less biased framing fosters both credulity and moral emotional reactions, which, in turn, affect the probability of sharing them online. In contrast, only in the loyalty domain, emotional activation bypasses the subject’s ability to correctly discriminate the veracity of the specific news. This result is in line with studies on the importance of political motivation in the credibility of misleading news [20,23], where the moral domain of loyalty tends to emotionally activate the readers—especially when the stimulus has a strong bias coherently with a highly polarized setting. In fact, in line with the research on political dualism, from our results, it emerges that tribal logic wins over the evaluative processing, since this is the only domain in which participants tend to share news framed with a high level of bias.

With respect to moral domains, however, the literature [19] also highlights how they are related to the individual dimension of personal values, by indicating how self-transcendence would mainly be associated with harm/care and fairness/reciprocity moral concerns, while conservation would be associated with authority/respect. Overall, present results suggest that conservation and self-transcendence values can play an opposite influence in the sharing process. High levels of self-transcendence can be considered a protective factor in the sharing of misleading news, except in the case of the care domain. Self-transcendence, on the other hand, prevents the sharing of misleading news concerning the violation of formalized norms (regulations), group conventions, and respect for authority. Differently, in the case of evident aggression/damage to a victim, people with high levels of self-transcendence activate moral emotions and the likelihood of misleading news sharing. This could be related to the importance of safeguarding “the wellbeing” of the alleged perpetrators of such violations before a clear and acknowledged guilt. Therefore, people with high self-transcendence are cautious in attacking/blaming, except in the case of victims of aggression, as represented in the domain of care.

A higher level of conservation values fosters the sharing of almost all moral violations, except those pertaining to the moral domain of care, where there are no significant relationships. Specifically, results suggest that, in the case of stimuli related to the violation of formal and informal rules that regulate behavior within their own society, people with high conservation tend to share them regardless of the cognitive evaluation of truthfulness.

In the case of the news stimuli about damaging behavior towards “earthly” authorities (e.g., the police) and “sacred” authorities (e.g., religious figures), people with high conservation are more likely to consider the low-bias misleading news more credible and become emotionally activated. Possibly, this is because, for people with high conservation, it is necessary to ensure the social order at a horizontal level: when it comes to maintaining the “hierarchical” order, they are more emotionally sensitive and cognitively permeable, so the sharing intention is directly related [15]. Concerning the limitations of the study, as this is pilot research, future studies will benefit from a larger and more diverse participant sample, including a higher variability of the participants’ age (e.g., teenagers/young people). Cultural factors will be taken into account in future works as well.

Concerning the relation between values and online sharing, we found, in some cases, a direct relation (in particular in fairness and loyalty), independently from cognitive and emotional processes; this can be due to less interest in those scenarios, but, in future studies, physiological measurements and reaction times will also be calculated when reading the news. Furthermore, the results highlight how people with good linguistic and communicative knowledge can better recognize the presence of high biases associated with moral misleading news, giving them less credibility and emotional activation. This limitation could be addressed by replicating the present study in different cultures and differentiated levels of linguistic or communicative knowledge, also, to obtain wider evidence showing the generalizability of the present findings. Skills related to internet browsing, use of social networks, as well as the frequency of use of digital communication tools can potentially have an impact on the credibility of the news; therefore, in future research using a methodology similar to the one presented in this study, it would certainly be desirable to investigate the digital skill levels of the sample. Moreover, the posited models should be replicated on larger and gender-balanced samples considering also the potential age-related differences.

Another limitation inherent in the level of control required by the quasi-experimental design is that of having presented a static screenshot of the news, stopping the information extracted from a precise social network (i.e., Instagram) at a specific moment of interaction. The undeniable importance of the unique affordances specific to the various social networks, as well as the presence of content recommendation methods and different automatic advertising models [56], prompts us to certainly want to overcome this limitation by considering in future studies different contexts based on multiple social networks in increasingly realistic and interactive ways.

With respect to the possible social and practical implications, these results suggest how important it can be to promote educational programs, especially considering youths and young adults, aimed at reinforcing linguistic competence, and also recognizing one’s own personal values and the associated potential vulnerability, in particular, considering the content of the misleading news. In fact, in the case of moral political communications, misleading news about violations of law, in-group interests, and national authorities could be more easily shared by subjects with high levels of conservation values.

The conservation values, such as security, tradition, and conformity, would promote motivated reasoning that confirms the credibility of this kind of news. Moreover, conservation values would make misleading news related to these domains more emotionally activating. This result is in line with other findings attesting that conservatives are more likely to believe false news [57]. Regarding this research field, we add and argue the importance of taking into account not only the political orientation but also the moral contents of biased news [58,59]. The interaction between communication contents and individual values can exacerbate negative reactions by hindering resistance to misinformation; people could further reinforce their own conservative moral principles by believing in overwhelming moral misleading news.

## Figures and Tables

**Table 1:** Descriptive statistics.

		Mean	SD	Sk	K
	Credibility	3.882	0.836	−1.272	2.376
Care	Moral emotions	3.252	0.905	−0.376	−0.482
	Online sharing	3.208	1.413	−0.376	−1.170
	Credibility	3.189	0.999	−0.410	−0.348
Fairness	Moral emotions	1.716	0.757	1.415	1.816
	Online sharing	2.155	1.234	0.815	−0.352
	Credibility	2.610	1.210	0.133	−1.051
Loyalty	Moral emotions	1.347	0.605	2.387	6.401
	Online sharing	1.492	0.926	2.198	4.831
	Credibility	3.113	1.080	−0.320	−0.709
Authority	Moral emotions	1.822	0.915	1.375	1.290
	Online sharing	2.100	1.275	0.836	−0.482
	Credibility	2.352	1.174	0.395	−1.046
Purity	Moral emotions	1.762	0.844	1.309	1.100
	Online sharing	2.023	1.279	0.905	−0.531

**Table 2:** Correlation matrix.

Moral Domain	Proximal Dimensions			Framing	Personal Values	
		CR	ME	BIASED	CONS	SELF-TR
Care	CR			0.255 **	0.230 **	0.158
	ME	0.473 **		0.230 **	0.302 **	0.376 **
	OS	0.467 **	0.461 **	0.200 *	0.127	0.049
Fairness	CR			0.341 **	0.254 **	0.141
	ME	0.359 **		0.187 *	0.241 **	0.190 *
	OS	0.330 **	0.428 **	0.154	0.247 **	−0.058
Loyalty	CR			0.174 *	0.125	0.041
	ME	0.162		−0.175 *	0.236 **	0.192 *
	OS	0.200 *	0.562 **	−0.018	0.206 *	−0.054
Authority	CR			0.426 **	0.340 **	0.237 **
	ME	0.491 **		0.081	0.347 **	0.232 **
	OS	0.354 **	0.363 **	0.175 *	0.225 *	0.003
Purity	CR			0.277 **	0.275 **	0.107
	ME	0.667 **		0.214 *	0.325 **	0.227 **
	OS	0.490 **	0.471 **	0.094	0.162	−0.022

*. Correlation is significant at the 0.05 level (2-tailed); **. Correlation is significant at the 0.01 level (2-tailed).

## Data Availability

The data that support the findings of this study are not openly available due to human data and are available from the corresponding author upon request in a controlled access repository.

## Appendix A. Stimuli Images

English translation: “It never rains but it pours: disabled man attacked on the wild streets of the capital. It happened last night on the streets of Rome to a 16-year-old boy, sitting on the steps of the Church of Santa Maria Maggiore. After getting up to reach the subway, the boy walked slowly towards the station having one of his two legs amputated, holding his crutches. His uncertain walk attracted the attention of two young men evidently intoxicated, who started to mock him in a blatant way triggering the alarmed reaction of the boy who started to cry for help to the unconcerned bystanders. The two young people were out of control, probably at the end of an evening of drinking and taking illegal substances, since they continued to bother the passers-by. The disabled boy shortly afterward managed to change his route, thus moving away from the two aggressors’’.

English translation: “When disability becomes a burden: young people insult a disabled person. It happened last light on the streets of Rome to a 16 years old boy, sitting on the steps of the Church of Santa Maria Maggiore. After getting up to reach the subway station, the boy walked slowly towards the station having one of his two legs amputated, holding his crutches. His uncertain walk attracted the attention of two young men who started to mock him, causing the boy to call for help. None of the passers-by took the disabled boy’s side, and he decided to change his direction and move away”.
